# Are pre-existing psychiatric disorders the only reason for involuntary holds in the emergency department?

**DOI:** 10.1186/s12245-020-0265-4

**Published:** 2020-02-03

**Authors:** Christian Lachner, Michael J. Maniaci, Tyler F. Vadeboncoeur, Nancy L. Dawson, Teresa A. Rummans, Archana Roy, Lorrina L. Hall, M. Caroline Burton

**Affiliations:** 1 Division of Psychiatry, Mayo Clinic, Jacksonville, FL USA; 2 Division of Hospital Internal Medicine, Mayo Clinic, 4500 San Pablo Rd, Jacksonville, FL 32224 USA; 3 Department of Emergency Medicine, Mayo Clinic, Jacksonville, FL USA; 4 Security and Transportation, Mayo Clinic, Jacksonville, FL USA; 5 Division of Hospital Internal Medicine, Mayo Clinic, Rochester, MN USA

**Keywords:** Involuntary hold, Psychiatric hold

## Abstract

**Objectives:**

To determine the role of previous psychiatric disorders including substance use disorders on emergency department (ED) patients on involuntary holds and compare presentations, treatment, and outcomes based on cause.

**Methods:**

We conducted a retrospective study of patients ≥ 18 years old on involuntary holds in the ED of a tertiary care center from January 1, 2013, to November 30, 2015. Demographic and clinical information were collected. Those with and without prior psychiatric disorder including substance use disorder were compared.

**Results:**

We identified 251 patients of which 129 (51.4%) had a psychiatric disorder, 23 (9.2%) had a substance use disorder, and 86 (34.3%) had both. Thirteen patients (5.2%) had no psychiatric disorder or substance use disorder and the majority 10 (76.9%) were on involuntary holds due to suicidal threats related to pain or another medical problem. Patients without a psychiatric or substance use disorder were older (55 years [17.8] vs 42 [19]; *P* = 0.01), more likely to be married (10 [76.9%] vs 64 [26.9%]; *P* < 0.001), and had more medical comorbidities (10 [76.9%] vs 114 [47.9%]; *P* = 0.049) compared with those without a psychiatric or substance use disorder.

**Conclusion:**

Patients on involuntary holds most commonly have pre-existing psychiatric disorder including substance use disorder. Patients on involuntary holds without history of psychiatric disorder often have severe pain or other active medical conditions which may contribute to suicidal thoughts. Addressing these underlying medical issues may be crucial in preventing further psychiatric decompensation.

## Introduction

Involuntary holds are commonly placed by emergency physicians and are often associated with longer emergency department (ED) length of stay [1, 2]. Much of what is known about involuntary holds comes from studies of patients admitted to psychiatric units for exacerbation of their ongoing psychiatric disorder including substance use disorder. Approximately 40% of patients with psychiatric disorder in the USA are untreated [3], and not uncommonly, these patients experience a mental health crisis requiring an emergency involuntary hold. Studies show that the majority of involuntarily committed patients have more severe psychiatric illness, including psychotic disorders, especially schizophrenia, higher rates of assaultive behaviors prior to admission, and lack of treatment adherence [4, 5].

While prior psychiatric disorder is typically associated with involuntary hold, there are other less common reasons for involuntary holds in the ED in patients with no prior psychiatric disorder. For example, patients with severe behavioral disturbances associated with cognitive impairment from dementia or other medical conditions may require an emergency involuntary hold. Every state in the USA and District of Columbia have laws that allow health care providers to revoke the civil liberties of patients suspected of having mental health problems who are unwilling or unable to seek care and may pose a danger to self or others [6]. These laws are meant to protect the patient and society from unintended consequences of untreated conditions that produce verbal or behavioral threats of violence. In Florida, the Baker Act provides for a 72-h emergency hold on patients with suspected mental illness posing a danger to self or others and refusing or lacking capacity to consent for care.

With the number of involuntary patients being seen in the ED increasing [7], it is important to understand the clinical and demographic characteristics of these patients in order to provide effective and efficient care. The objectives of this study are to determine the role of previous psychiatric disorder including substance use disorder on patients in the ED of an acute care hospital on involuntary holds and compare demographic and clinical presentations, treatment, and outcomes based on cause.

## Materials and methods

This retrospective chart review study, deemed exempt by the Mayo Clinic Institutional Review Board, was conducted at a suburban academic tertiary care center in Florida that also serves the community and has an annual ED volume of 27,305 patient visits. Adults 18 years and older presenting or placed on involuntary holds in the ED from January 1, 2013, through November 30, 2015, were included in this study. If a patient had multiple admissions during the study period, only the first admission was included. Specific demographic information collected included age, race, sex, marital status, housing, and payer source. Clinical data collected included reason for involuntary holds, prior psychiatric or substance use disorders, suicide attempts, medical condition, violence in the ED, length of stay (LOS), disposition, and 30-day readmission.

Study data were collected and managed using REDCap (Research Electronic Data Capture) [8]. REDCap is a secure, web-based application designed to support data capture for research studies, providing (1) an intuitive interface for validated data entry, (2) audit trails for tracking data manipulation and export procedures, (3) automated export procedures for seamless data downloads to common statistical packages, and (4) procedures for importing data from external sources.

Standard descriptive statistics were used for patient demographics and clinical factors. The two patient cohorts were compared using the *χ*^2^ test and Fisher exact test to evaluate categorical variables and Wilcoxon rank sum test to evaluate continuous variables. *P* less than or equal to .05 was considered statistically significant. All statistical analyses were performed using the SAS version 9.3 software (SAS Institute Inc).

## Results

There were 278 patients on involuntary holds during the study period. Eleven were readmissions during the study period and were excluded leaving 267 unique patients. Sixteen were excluded because of age less than 18 leaving 251 patients in the study population. Of the 251 patients studied, only 13 patients were found to have no prior history of psychiatric disorder. Univariate analyses of patient demographics are shown in Table 1. Patients without a psychiatric or substance use disorder were older (median age, 55 vs 42 years; *P* = .01) and were more likely to be married (76.9% vs 26.9%; *P* < 0.001). There was no significant difference in gender, race, housing status, or payer status (Table 1).

**Table 1:** Demographics of patients with psychiatric disorder or substance abuse

Covariate	No, *N* = 13	Yes, *N* = 238	*p* value
Patient characteristics			
Age (at time of Baker Act)			0.01^†^
Median (Q1, Q3)	55 (48, 67)	42 (28, 53)	
Range	20, 91	18, 94	
Gender			0.82^‡^
Female	7 (53.8%)	136 (57.1%)	
Male	6 (46.2%)	102 (42.9%)	
Race			0.29^§^
White	9 (69.2%)	193 (81.1%)	
Non-White	4 (28.6%)	45 (18.9%)	
Marital status			< 0.001^§^
Married	10 (76.9%)	64 (26.9%)	
Not married	3 (23.1%)	174 (73.1%)	
Housing			0.98^§^
Missing	0 (0.0)	11 (4.6)	
Own/rent house/apartment	12 (92.3%)	197 (82.8%)	
Homeless/shelter/institution/living in another’s home	1 (7.7%)	30 (12.6%)	
Payer			0.07^§^
Self-pay	1 (7.7%)	81 (34.0%)	
All others	12 (92.3%)	157 (66.0%)	

^†^Wilcox rank sum test

^‡^Chi-square

^§^Fisher exact

Of the 251 patients, 129 (51.4%) had a psychiatric disorder, 23 (9.2%) had only substance use disorder, 86 (34.3%) had both substance use and psychiatric disorders, and 13 (5.2%) had no prior history of psychiatric or substance use disorders. Univariate analyses of patient clinical characteristics were performed, and patients without a psychiatric or substance use disorder were found to have significantly more medical problems (76.9% vs 47.9%; *P* = .05). There was no significant difference in whether the patient demonstrated violent behavior in the ED, had suicidal or homicidal ideation, frequency of serum alcohol and urine drug testing, ED LOS, ED discharge disposition, whether the involuntary hold was removed in the ED, 30-day readmissions, or if the patient required hospital admission (Table 2).

**Table 2:** Characteristics of patients with psychiatric disorder including substance abuse

Covariate	No, *N* = 13	Yes, *N* = 238	*p* value
Psychiatry and drug information			
Current psychiatric disorder only	0 (0.0%)	129 (51.4%)	< 0.001^§^
Current drug abuse only (other than tobacco)	0 (0.0%)	23 (9.2%)	< 0.001^§^
Current both psychiatric and drug abuse	0 (0.0%)	86 (34.3%)	< 0.001^§^
Neither psychiatric and drug abuse	13 (5.2%)	0 (0.0%)	< 0.001^§^
Medical information			
Does the patient have medical issues?	10 (76.9%)	114 (47.9%)	0.05^§^
Medical diagnoses, diabetes mellitus	1 (7.7%)	15 (6.3%)	0.58^§^
Medical diagnoses, hypertension	3 (23.1%)	39 (16.4%)	0.46^§^
Medical diagnoses, liver disease	0 (0.0%)	3 (1.3%)	1.00^§^
Medical diagnoses, coronary artery disease	1 (7.7%)	6 (2.5%)	0.31^§^
Medical diagnoses, COPD	0 (0.0%)	5 (2.1%)	1.00^§^
Medical diagnoses, other	10 (76.9%)	102 (42.9%)	0.02^§^
Violence information			
Patient was violent in the ED	0 (0.0%)	22 (9.2%)	0.62^§^
Reason for involuntary hold			
Suicidal	10 (76.9%)	174 (73.1%)	0.52^§^
Homicidal	0 (0.0%)	13 (5.5%)	1.00^§^
Danger to self (not suicidal)	2 (15.3%)	72 (5.5%)	0.76^§^
Mental illness	1 (7.7%)	64 (5.5%)	0.20^§^
Drug information			
Serum alcohol level obtained?	7 (53.8%)	174 (73.1%)	0.132^†^
Blood alcohol concentration (mg/dL)			0.042^†^
*N*	7	174	
Median (Q1, Q3)	0 (0.0, 0.0)	0.0 (0.0, 201.6)	
Range	0.0, 0.0	0.0, 479.3	
Urine drug screen	9 (69.2%)	189 (79.4%)	0.482^§^
Drug in urine, amphetamine	0 (0%)	11 (4.6%)	1.000^§^
Drug in urine, methamphetamine	0 (0%)	0 (0%)	–
Drug in urine, barbiturate	0 (0%)	8 (3.4%)	1.000^§^
Drug in urine, benzodiazepine	3 (23.1%)	52 (21.8%)	1.000^§^
Drug in urine, cocaine	1 (7.7%)	19 (8.0%)	1.000^§^
Drug in urine, opiate	2 (15.4%)	37 (15.5%)	1.000^§^
Drug in urine, PCP	0 (0.0%)	1 (0.4%)	1.000^§^
Drug in urine, THC	0 (0.0%)	35 (14.7%)	0.225^§^
Drug in urine, tricyclic antidepressant	0 (0.0%)	15 (6.3%)	1.000^§^
Drug in urine, none	5 (38.5%)	78 (32.8%)	0.671^‡^
ED stay information			
Emergency department length of stay (hours)			0.45^†^
Median (Q1, Q3)	6 (5, 10)	6 (5, 11)	
ED discharge disposition			0.06^§^
Missing	1 (7.7%)	36 (15.1%)	
Home	2 (15.4%)	5 (2.1%)	
Inpatient psychiatric/treatment facility	10 (76.9%)	197 (82.7%)	
Involuntary hold removed in the ED	1 (7.7%)	7 (2.9%)	0.37^§^
ED readmission within 30 days	1 (7.7%)	12 (5.0%)	0.53^§^
Hospital admission information			
Patient was admitted to the hospital	1 (7.7%)	36 (15.1%)	0.70^§^

^†^Wilcoxon

^‡^Chi-square

^§^Fisher exact

Of those 13 patients without a psychiatric disorder, 4 (30.8%) showed self-harming behaviors (3 overdosed, 1 had a self-inflicted wound). Three patients (23.1%) presented with psychotic symptoms (delusions): 1 was tapering opiates; 1 had persecutory delusions in the setting of altered mental status; and 1 had persecutory delusions worsening over several months in the setting of abdominal pain, chronic menorrhagia, and thyroid disease. The remaining 6 patients reported suicidal ideations in the setting of pain or a significant medical condition (Table 3).

**Table 3:** Demographic and clinical variables of patients without history including psychiatric or substance use disorders

Age (years)	Race	Sex	Marital status	Medical insurance	Medical problem	Reason for involuntary hold	Involuntary hold placed in ED?
80	White	Male	Married	Medicare	Brain tumor	Suicidal	Yes
91	White	Male	Married	Medicare	Back pain, OD	Suicidal	No
49	Black	Female	Married	Medicaid	History of brain aneurysm surgery, seizures	Suicidal	Yes
38	Black	Female	Married	Medicaid	Self-inflicted wound	Self-harm	No
67	White	Female	Married	Commercial	Back pain	Suicidal	No
59	Asian	Female	Married	Commercial	ESRD, sepsis	Suicidal	No
56	White	Male	Divorced	Medicare	Flank pain, renal cell carcinoma history of nephrectomy	Suicidal	Yes
50	White	Male	Married	Commercial	Altered mental status	Transient psychosis	No
20	White	Female	Single	Commercial	Fibromyalgia	Suicidal	Yes
55	White	Male	Married	Commercial	OD after court sentence	Suicidal	No
71	White	Male	Married	Medicare	Neuropathic pain, benzodiazepines, and opiates in urine	Transient psychosis, suicidal	Yes
40	Hispanic	Female	Married	Commercial	Abdominal pain, chronic menorrhagia, thyroid problems	Paranoia, auditory hallucinations	Yes
48	White	Female	Single	Self-pay	Severe asthma, OD on gabapentin	Suicidal	No

*ED* emergency department, *ESRD* end-stage renal disease, *OD* overdose

Of those 13 patients without a psychiatric disorder, 10 (76.9%) had a documented previous medical comorbidity. All 13 patients presented to the ED with a recent or acute medical problem. Seven of the 13 patients (53.8%) reported pain as their major medical problem: 2 patients had back pain, 1 had abdominal pain related to chronic menorrhagia, 1 had flank pain after nephrectomy due to cancer, and 3 patients had neuropathic pain. Of the remaining 6 patients, 1 had brain surgery, 1 had a brain tumor, 1 was septic in the setting of chronic kidney disease, 1 had a self-inflicted wound, 1 had severe asthma, and 1 had recently overdosed after receiving a court sentence. Six patients (46.2%) were placed on involuntary hold in the ED, and 7 (53.8%) prior to arrival in the ED. The most common reason for involuntary hold was suicidal ideation in 10 patients (76.9%), which 5 (38.5%) attributed to pain. Psychosis was present in the remaining 3 patients (Table 1).

## Discussion

To our knowledge, this is the first study to examine the underlying reasons for involuntary holds on patients in the ED while examining the differences between those with and without psychiatric disorder including substance use disorder. One of the major findings was that 95% of involuntary hold patients in the ED had a psychiatric disorder. This finding emphasizes the importance of having ED staff trained in recognizing and treating acute psychiatric illnesses. Having access to a clinical psychiatrist may be crucial in order to expedite proper diagnosis and avoid delay in therapy. Likewise, recognizing and reversing the effects of substance abuse remain a critical part of ED care in this patient population.

We found that only a small percentage of patients (5%) did not have a prior history of psychiatric disorder. This group of 13 patients had significantly more medical comorbidities (76.9% vs 47.9%), and all had an active medical problem upon presentation to the ED, underscoring the importance of having well-trained staff and an adequate infrastructure in order to deliver proper medical care to a patient population that by definition poses a threat to self or others (involuntary holds). Comorbid medical conditions are a known source of psychological distress [9], especially when the comorbid condition is producing pain and the association between chronic disease and depression is well established [10]. Further, many long-term care patients often have behavioral health disorders related to chronic medical conditions [11]. We found that none of the 13 patients acted out violently, which may reflect intermittent psychological distress as a consequence of medical comorbidity and not behavior associated with substance use or other chronic psychiatric disorder. It is also possible that even though the patient’s involuntary status may suggest a true unwillingness to receive care, some of these patients are willing to cooperate once in the ED; clinicians should continue their involuntary status given the risks associated with recent disclosure of suicidal ideations in order to guarantee a safe environment for care, rather than leaving this highly important endeavor and risk up to the patient. Seven of these patients (53.8%) presented to the ED voluntarily, which underscores the importance of having staff trained to recognize safety concerns in patients who may not otherwise be at risk for a mental health crisis.

In over half of these patients (53.8%), the active problem involved ongoing pain. It was the uncontrolled medical problems and pain that contributed to development of suicidal ideations and/or attempts in the majority of the patients. In fact, in more than one third of these patients, suicide was reported as a means to end physical suffering. This finding may have implications in terms of better addressing pain in the medically ill population in order to prevent hopelessness and helplessness that often lead to suicidal ideations. Pain is a known risk factor for mental health problems, including suicidal ideation [12], and comorbid medical conditions have been reported as a significant risk factor for suicide [13]. Pain is also a recognized risk factor for depression, which reinforces the case for possible undiagnosed depression affecting this patient population and the need to screen medical populations for depressive symptoms [14].

Additional information found on the patients without a history of psychiatric disorder were that they were older, more likely to be married, and had more medical comorbidities than those who did. This cohort appeared socioeconomically and psychologically more stable; nevertheless, these patients similarly suffered a mental health crisis. Gender, race, housing status, payer status, and presence of suicidal or homicidal ideations were not different between this group and those with psychiatric or substance abuse problems. This may suggest that patients presenting to the ED on emergency holds without having psychiatric disorders or substance abuse are more homogeneous than different to patients with mental health or substance abuse, raising important points. Traditionally, risk of harm to self or others has been attributed to psychiatric patients or those with specifically substance abuse problems, but given these findings, all patients admitted on involuntary holds carried a similar risk of harm. Important implications of these findings could suggest the need to review safety policies and infrastructure for all emergency personnel who cares for patients on involuntary holds, independently of their mental health or substance abuse status.

## Limitations

As this is a retrospective study, there are inherent limitations. These include inconsistencies in reporting of prior history of psychiatric disorder including substance use disorder. Also, this study was conducted at a single site in a suburban location with a more ethnically homogeneous patient population than an intercity hospital in a large, metropolitan area, which limits generalizability of our findings. Education and employment information was not included in the medical record for a number of patients, which limited our ability to use these variables in our analysis. Cohorts were not compared with respect to ED diagnostic work-up and treatment. We were unable to review medical records of patients transferred for inpatient psychiatric care; consequently, final disposition and diagnostic determinations were unknown. Finally, although not unexpected, the small number of patients without a history of psychiatric or substance use disorder limited our ability to show an association between demographic and clinical variables and involuntary hold.

## Conclusions

Patients on involuntary holds most commonly have a prior history of psychiatric disorder including substance use disorder. The majority of patients who did not have history of psychiatric disorder were suicidal in the setting of pain and other medical conditions. This may represent a growing population as our society ages and more people have devastating medical conditions. Consequently, as more people come to ED, providers may increasingly encounter medically ill patients with severe psychiatric symptoms warranting involuntary holds. It is imperative these patients first undergo comprehensive evaluation to stabilize and/or treat medical problems before referral for inpatient psychiatric treatment.

## Data Availability

All datasets used and/or analyzed during the current study are available from the corresponding author upon reasonable request.
